# Therapeutic Efficacy of HPβ‐CD‐Angiotensin‐(1‐7) Oral Formulation in Muscle Injury Recovery in Rat

**DOI:** 10.1111/jcmm.70851

**Published:** 2025-09-22

**Authors:** Nádia Lúcia Totou, Ana Maria Sampaio Rocha, Samara Silva de Moura, César Henrique Pereira, Fabricio Sampaio Coelho, Douglas Daniel Dophine, Daniel Barbosa Coelho, Emerson Cruz de Oliveira, Robson Augusto Souza dos Santos, Lenice Kappes Becker, Wanderson Geraldo de Lima

**Affiliations:** ^1^ Biological Sciences Research Center—Postgraduate Program in Biological Sciences Federal University of Ouro Preto Ouro Preto Brazil; ^2^ School of Physical Education Federal University of Ouro Preto Ouro Preto Brazil; ^3^ Department of Physiology and Biophysics, Institute of Biological Sciences Federal University of Minas Gerais Belo Horizonte Brazil

**Keywords:** angiotensin, connective tissue growth factor, fibrosis, inflammation, muscle injury

## Abstract

To evaluate the therapeutic effect of oral treatment with HPβ‐CD‐angiotensin‐(1‐7) (Ang‐(1‐7)) on muscle recovery after laceration injury. Wistar rats were divided into four groups: Control (*n* = 10); HPβ‐CD‐Ang‐(1‐7) (*n* = 10); muscle injury + HPβ‐CD (MI + Placebo) (*n* = 24); and muscle injury + HPβ‐CD‐Ang‐(1‐7) (MI + Ang‐(1‐7)) (*n* = 24). After 7–21 days of treatment, physical performance, histological features and the expression of pro‐ and anti‐fibrotic genes were evaluated. The MI + Ang‐(1‐7) group showed improved control of the inflammatory phase and reduced deposition of collagen types I and III compared to MI + Placebo. CTGF gene expression analysis revealed lower levels of pro‐fibrotic markers and higher expression of proteins involved in blocking fibrotic pathways. In treadmill tests, MI + Ang‐(1‐7) animals also showed superior physical performance at all evaluated time points. Oral treatment with Ang‐(1‐7) is effective in promoting recovery from muscle injuries, particularly fibrotic lesions, while preserving muscle function and enhancing physical performance.

## 1 Introduction

Due to its superficial location, muscular tissue is susceptible to various forms of traumatic injuries that can result in fibre necrosis [1]. In 90% of cases, such injuries can arise from contusion, strain and/or muscular laceration [2]. In certain instances, these injuries may exhibit a slow repair process, along with a high recurrence rate, leading to more severe conditions than the initial injury [3].

Treatment strategies for muscular injuries are crucial and can range from simple treatments like local ice application to the requirement of specific medication use. In this context, previous studies [4, 5, 6, 7, 8] have been conducted with the objective of achieving morpho‐functional recovery of muscular tissue by promoting minimal scar tissue formation, thereby preventing the emergence of new injuries and facilitating a swift return to physical activities. The identification of safe and effective strategies for managing muscular injury remains a challenge with relevant clinical potential.

Other studies have demonstrated that both classical components of the renin–angiotensin system (RAS), such as angiotensin II (Ang II), AT1 receptor and angiotensin‐converting enzyme (ACE), as well as newer components like ACE2, angiotensin‐(1‐7) (Ang‐(1‐7)), and its Mas receptor, have been identified within skeletal striated muscular tissues. This suggests the presence of a locally active RAS within skeletal musculature [9, 10]. Recent investigations indicate that the protective axis of the RAS (Ang‐(1‐7)/ACE2/Mas) exerts beneficial effects on skeletal musculature, including improved insulin sensitivity [11] and anti‐atrophic factors [9, 12, 13].

The role of Ang‐(1‐7) has been studied in skeletal musculature [14, 15, 16]. This peptide has significant effects on muscular remodelling observed across various models, including the infusion of Ang II [9, 10, 17], muscular dystrophy [18, 19] disuse atrophy [20, 21] and exhaustive physical exercise [12, 22].

In a previous study, animals treated with the oral formulation of HPβ‐CD‐angiotensin‐(1‐7) exhibited inflammatory responses and fibrosis induction comparable to those of the control group after undergoing a physical exercise protocol involving eccentric contractions, which are known to compromise skeletal muscle integrity [12]. Skeletal muscle injuries are typically stratified into three categories: mild, moderate and severe [23]. Laceration is classified as a severe injury, characterised by marked loss of skeletal muscle function [23]. Several studies have demonstrated that the laceration model provides a more reliable approach for evaluating muscle repair and for investigating granulation tissue deposition [24, 25]. The present study advances this line of research by evaluating the effects of HPβ‐CD‐angiotensin‐(1‐7) using the laceration injury model.

Effective strategies for treating muscular injuries still need to be identified, given that Ang‐(1‐7) plays a significant role in muscular inflammation and fibrosis. Therefore, the aim of the present study was to assess the effect of oral administration of Ang‐(1‐7) formulation in the treatment of inflammatory and fibrotic lesions in the skeletal striated muscles of Wistar rats that had undergone laceration‐induced injuries.

## 2 Materials and Methods

### 2.1 Ethical Approval

The procedures were approved by the Animal Research Ethics Committee of the Federal University of Ouro Preto to evaluate the efficiency of oral Angiotensin‐(1‐7) formulation in muscle repair. Functional (maximal test), histopathology (inflammation, collagen type) and RNA expression were assessed.

### 2.2 Animals

Male Wistar rats (*Rattus norvegicus*), weighing between 250 and 300 g (inclusion criteria), provided by the Animal Science Center of the Federal University of Ouro Preto, were kept under identical conditions with a 12/12‐h light–dark cycle, receiving water and food ad libitum. The sample size was obtained using parameters from previous studies of the group. For the calculation, an online sample calculator was used. The animals were daily monitored; no animals showed signs of distress.

### 2.3 Experimental Groups

The animals were randomly divided into four experimental groups using a random number generator in Excel 2003 (Microsoft Corporation, Redmond, WA, USA): Control (*n* = 10) animals kept under the same environmental conditions without undergoing surgical procedures or any form of treatment; HPβ‐CD‐angiotensin‐(1‐7) (Ang‐(1‐7)) (*n* = 10) animals without surgical procedures but treated with HPβ‐CD Angiotensin‐(1‐7); muscular injury + HPβ‐CD (MI + Placebo) (*n* = 24) animals treated only with HPβ‐CD (inclusion complex) and muscular injury + HPβ‐CD‐angiotensin‐(1‐7) (MI + Ang‐(1‐7)) (*n* = 24) subjected to a muscle laceration injury protocol and treated with an oral formulation of HPβ‐CD Angiotensin‐(1‐7).

### 2.4 Muscular Injury Laceration Protocol

The laceration model was previously described [26] and was adapted to the gastrocnemius skeletal muscle of rats. Anaesthesia was induced in restrained rats through intraperitoneal injection of ketamine (80 mg/kg) and xylazine (10 mg/kg). Following deep anaesthesia, a longitudinal skin incision was made in the posterior calf region, and subcutaneous dissection exposed the gastrocnemius skeletal muscle. The gastrocnemius skeletal muscle was lacerated at 60% of its insertion length, encompassing 50% of its width and 50% of its thickness. Tramadol (5 mg/kg) was administered intraperitoneally immediately following muscle laceration. This procedure was repeated for three consecutive days, with the aim of reducing the animal's pain and suffering.

### 2.5 Oral Formulation of HPβ‐CD Angiotensin‐(1‐7) Treatment

Angiotensin‐(1‐7) included in hydroxy‐propyl‐beta‐cyclodextrin (HPβ‐CD) was administered as a single daily oral dose (gavage) until euthanasia. The treatment was conducted in two distinct time periods: One group received treatment for 7 days, and another group for 21 days. The treatment solutions were based on previous studies, with a dose of 50 μg/kg body mass [27]. The placebo solution (HPβ‐CD) was formulated similarly to the treatment solution, excluding Angiotensin‐(1‐7). The final gavage volume [HPβCD and HPβCD/Ang‐(1‐7)] was approximately 0.5 mL. At the beginning of the experiments, the animals body mass was measured to ensure group homogeneity and for the calculation of gavage volume. These procedures were repeated weekly until the end of the experiment. The animals were separated into cages according to the type of treatment. This strategy was used to minimise possible confusion in the administration of treatment solutions.

### 2.6 Maximal Physical Effort Test

Physical tests were carried out on the final day of oral treatment by an investigator blinded to treatment allocation. Animals underwent a maximal physical effort test on a treadmill. The animals underwent an adaptation process of running on the treadmill for 5 days at a constant speed of 15 m/min for 5 min per day. Failure to adapt to the treadmill was used as an exclusion criterion from the study. No animals were excluded during the adaptation period. For the effort test, the animals ran on the treadmill with an initial speed of 10 m/min (no incline), following a stepped protocol with speed increments of 3 m/min every 3 min until the animals reached their maximum sustainable speed. The effort test concluded when animals exhibited signs of exhaustion. Exhaustion was defined as the moment when the rat could no longer run within the rolling area, despite the treadmill speed increment, and remained outside the area for more than 10 s [28]. Another parameter evaluated was the number of failures, defined as the time that the animal spent outside the rolling area for < 10 s [29].

### 2.7 Euthanasia and Material Collection

Animals subjected to the laceration protocol and control group animals were divided into groups based on the time of euthanasia: 7–21 days post‐surgery. During euthanasia, the entire extent of the gastrocnemius skeletal muscle was excised and divided into two fragments. One part was stored in buffered formalin, and the other part was stored in a freezer at −80°C.

### 2.8 Histopathologic Assessments

After complete excision of the gastrocnemius muscle, the tissue was fixed in buffered formalin solution. Subsequently, tissues underwent routine histological processing. Paraffin sections of 4 μm thickness were placed on glass slides and stained with haematoxylin and eosin (HE) to evaluate tissue inflammatory infiltration. To determine the inflammatory pattern, 20 random images were taken from each rat at 440× magnification (total area: 1.49 × 106 μm^2^) using a Leica BM5000 microscope with a digital camera (Leica DFC 300 FX, Leica Microsystems, Wetzlar, Germany), linked to the RGB‐activated module and image capture software, Leica Application Suite. Total cells were quantified using Leica QWin Plus software by counting the total cell nuclei present in each image. Picrosirius red staining was employed to evaluate tissue collagen under polarised light optical microscopy. Quantitative analyses of type I and III collagen were performed. Type I collagen fibres exhibited strong birefringence in red or yellow, while type III collagen fibres showed primarily fine green fibrils with greenish birefringence.

### 2.9 Gene Expression Analysis by RT‐qPCR

Messenger RNA (mRNA) from cells of the gastrocnemius skeletal muscle tissue was obtained using TRIzol reagent (Invitrogen) as per the manufacturer's protocol. Complementary DNA (cDNA) was synthesised from 10 μg of RNA using Applied Biosystems Kit components. Real‐time quantitative PCR (RT‐qPCR) was performed using SYBR Green PCR master mix (Applied Biosystems) on a 7500 Fast Real‐Time PCR System (Applied Biosystems). The following primer sequences were used: Smad7: 5′‐GCGACGAAGAAGAGAAATGGG‐3′ and 5′‐AGGGAGGGAGGAATGGTGA‐3′; CTGF: 5′‐CAGGCTGGAGAAGCAGAGTCGT‐3′ and 5′‐CTGGTGCAGCCAGAAAGCTCAA‐3′; HPRT1: 5′‐GTCAAGCAGTACAGCCCCAA‐3′ and 5′‐GGCCTGTATCCAACACTTCG‐3′. To investigate the anti‐fibrotic effect of Angiotensin‐(1‐7), gene expression analysis of Smad7 in the gastrocnemius skeletal muscle of animals subjected to muscle laceration protocol and control groups was performed.

### 2.10 Statistical Analysis

Statistical analysis was conducted using GraphPad Prism software (version 8.0, San Diego, USA). A normality test was performed for each result. For statistically evaluating data with normal distribution, ANOVA was used, followed by Tukey's post hoc test. For all other non‐normally distributed data, the non‐parametric Kruskal–Wallis test was used, followed by Dunn's post. The reported values are presented in boxplots. The middle line within each boxplot represents the median value, and the whiskers indicate the minimum and maximum values. The lower and upper boundaries of each box represent the 25th and 75th percentiles of the sample. The significance level of *p* < 0.05 was considered statistically significant.

## 3 Results

### 3.1 Animal Body Mass

From the fourth week onwards, the animals in the MI + Ang‐(1‐7) group showed reduced weight gain (86.3 ± 4.5 g) compared to the MI + Placebo group (117.6 ± 2.6 g) (*p* < 0.0001) and to the Control group (108.2 ± 7.0 g) (*p* < 0.0001).

### 3.2 Maximal Physical Effort Test

**FIGURE 1:**
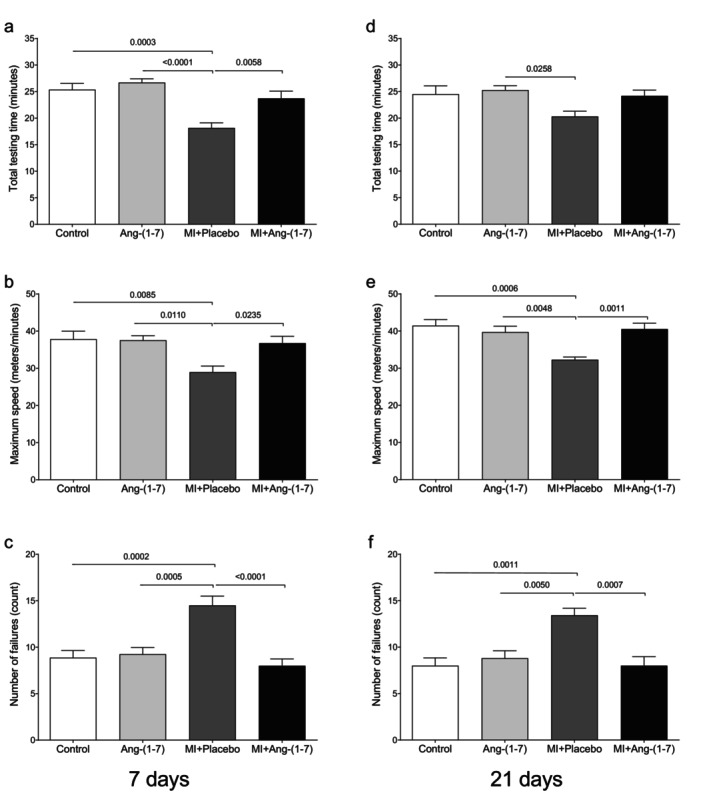
The treadmill running test in the four experimental groups was carried out after 7 days (a–c) or 21 days (d–f) of HPβ‐CD‐Ang‐(1‐7) treatment. Total testing time (min) (a and d); maximum speed reached (meters/min) (b and e); number of failures (count) (c and f). One‐way ANOVA followed by Tukey's multiple comparisons post‐test showed significant differences in the post‐test graphs; values are expressed as mean ± standard error of the mean. MI + Placebo showed significantly lower or higher values compared to the overall groups. The only exception was for total test time after 21 days (d), where the difference was only in relation to Ang‐(1‐7).

After 7 or 21 days of treatment, the MI + Placebo group showed significantly different values compared to the overall groups. The only exception was for total test time after 21 days (Figure 1d), where the difference was only in relation to Ang‐(1‐7). Multiple comparisons tests did not show significant differences among other groups (Figure 1a–f).

### 3.3 Cell Infiltration

**FIGURE 2:**
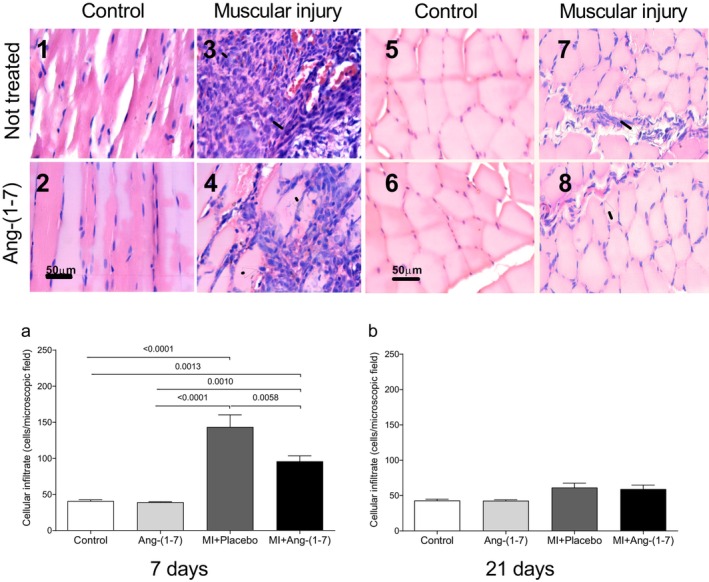
Number of inflammatory cells in the gastrocnemius muscle in the four experimental groups, after 7 days (1–4) or 21 days (5–8) of HPβ‐CD‐Ang‐(1‐7) treatment. Note the histological appearance compatible with normality in animals from the Control (photomicrographs 1 and 5) and Ang‐(1‐7) (photomicrographs 2 and 6) groups, and an increase in inflammatory cells (arrow) in animals from the MI + Placebo (photomicrographs 3 and 7) and MI + Ang‐(1‐7) groups (photomicrographs 4 and 8). The bar corresponds to 50 μm wide. After 7 days, only the Control and Ang‐(1‐7) groups were equal; overall post‐test comparisons were different, with *p* values shown in the graphs. The MI + Placebo vs. MI + Ang‐(1‐7) comparison is notable because of the significant differences (*p* = 0.0058). After 21 days, ANOVA indicates differences (*p* = 0.0175), but Tukey's multiple comparisons post‐test was not able to indicate which groups were different. One‐way ANOVA followed by Tukey's multiple comparisons post‐test; values are expressed as mean ± standard error of the mean.

The muscle laceration protocol was effective; an elevated inflammatory pattern was observed in the MI + Placebo and MI + Ang‐(1‐7) groups, while the Control and Ang‐(1‐7) groups remained normal. After 7 days, only the Control and Ang‐(1‐7) groups were equal; overall post‐test comparisons were different (*p* values in graphs, Figure 2a,b). The MI + Placebo vs. MI + Ang‐(1‐7) comparison showed significant differences (*p* = 0.0058) (Figure 2a). After 21 days, ANOVA indicated differences (*p* = 0.0175), but Tukey's multiple comparisons post‐test did not specify which groups were different (Figure 2b).

### 3.4 Collagen Deposition Analysis

**FIGURE 3:**
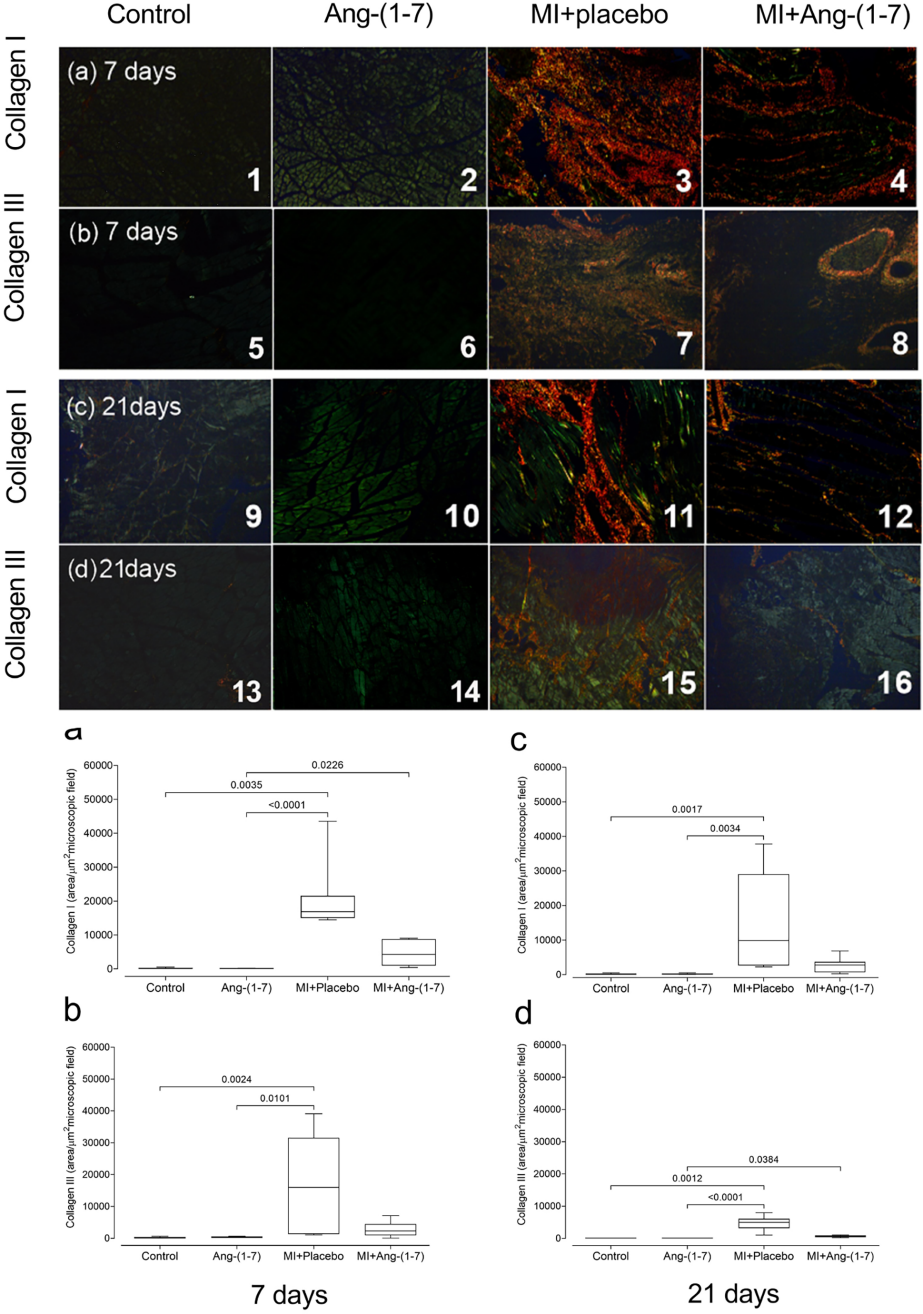
Area corresponding to the deposition of type I collagen (1–4 and 9–12) and type III collagen (5–8 and 13–16) in the gastrocnemius muscle in the four experimental groups, after 7 days (1–8) or 21 days (9–16) of HPβ‐CD‐Ang‐(1‐7) treatment. Representative photomicrographs of histological sections of gastrocnemius muscle stained using the Picrosirius Red technique observed under polarised light. Note the histological appearance compatible with normality in animals from the Control (photomicrographs 1, 5, 9 and 13) and Ang‐(1‐7) (photomicrographs 2, 6, 10 and 14) groups, and increased deposition of type I and III collagen in animals from the MI + Placebo (photomicrographs 3, 7, 11 and 15) and MI + Ang‐(1‐7) groups (photomicrographs 4, 8, 12 and 16). The bar corresponds to 100 μm wide. Kruskal–Wallis test with Dunn's multiple comparisons post‐test; whiskers represent minimum to maximum. In (a), MI + Placebo had the highest value, and post‐test *p* differences were as follows: Control vs. MI + Placebo (*p* = 0.0035); Ang‐(1‐7) vs. MI + Placebo (*p* < 0.0001); Ang‐(1‐7) vs. MI + Ang‐(1‐7) (*p* = 0.0226). In (b), MI + Placebo had the highest value, and post‐test *p* differences were as follows: Control vs. MI + Placebo (*p* = 0.0024); Ang‐(1‐7) vs. MI + Placebo (*p* = 0.0101). In (c), MI + Placebo had the highest value, and post‐test *p* differences were as follows: Control vs. MI + Placebo (*p* = 0.0017); Ang‐(1‐7) vs. MI + Placebo (*p* = 0.0034). In (d), MI + Placebo had the highest value, and post‐test *p* differences were as follows: Control vs. MI + Placebo (*p* = 0.0012); Ang‐(1‐7) vs. MI + Placebo (*p* < 0.0001); Ang‐(1‐7) vs. MI + Ang‐(1‐7) (*p* = 0.038).

Histological analysis of gastrocnemius muscle revealed significant alterations in collagen deposition following muscular injury. For type I collagen deposition at 7 days post‐treatment, the MI + Placebo group demonstrated significantly higher values compared to both the Control (*p* = 0.0035) and Ang‐(1‐7) (*p* < 0.0001) groups, while the MI + Ang‐(1‐7) group showed significantly greater deposition than the Ang‐(1‐7) group (*p* = 0.0226) but did not differ significantly from the Control group (Figure 3a). Similarly, type III collagen deposition at 7 days was significantly elevated in the MI + Placebo group compared to both Control (*p* = 0.0024) and Ang‐(1‐7) (*p* = 0.0101) groups, with no significant difference observed between MI + Ang‐(1‐7) and Control groups (Figure 3b). At 21 days, type I collagen deposition remained significantly higher in the MI + Placebo group compared to Control (*p* = 0.0017) and Ang‐(1‐7) (*p* = 0.0034) groups, with the MI + Ang‐(1‐7) group showing no significant difference from the Control group (Figure 3c). For type III collagen at 21 days, the MI + Placebo group maintained significantly elevated deposition compared to Control (*p* = 0.0012) and Ang‐(1‐7) (*p* < 0.0001) groups, while the MI + Ang‐(1‐7) group showed significantly higher deposition than the Ang‐(1‐7) group (*p* = 0.038) but remained statistically similar to the Control group (Figure 3d).

### 3.5 Reverse Transcription and Real‐Time PCR

**FIGURE 4:**
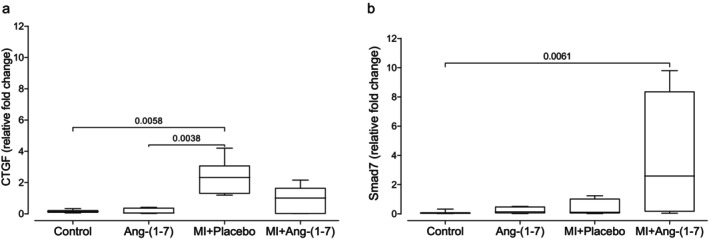
Gene expression of connective tissue growth factor (CTGF, a) and Smad7 protein (b) in the four experimental groups. Kruskal–Wallis test with Dunn's multiple comparisons post‐test; whiskers represent minimum to maximum. In (a), MI + Placebo had the highest value, and post‐test *p* differences were as follows: Control vs. MI + Placebo (*p* = 0.0058); Ang‐(1‐7) vs. MI + Placebo (*p* = 0.0038). In (b), MI + Ang‐(1‐7) had the highest value, and post‐test *p* differences were as follows: Control vs. MI + Ang‐(1‐7) (*p* = 0.0061).

Gene expression of connective tissue growth factor in the gastrocnemius skeletal muscle tissue was higher (relative fold change) in the MI + Placebo group compared to the Control and Ang‐(1‐7) groups, while the MI + Ang‐(1‐7) group presented intermediate values, being equal to all groups (Figure 4a). Conversely, Smad7 expression was higher (relative fold change) in the MI + Ang‐(1‐7) group compared to the Control group. The Control group presented values equal to the Ang‐(1‐7) and MI + Ang‐(1‐7) groups (Figure 4b).

## 4 Discussion

Experimental studies on muscle injury and repair models are essential for understanding biochemical and morphological mechanisms of tissue repair and functional recovery, as well as discovering novel strategies for treating muscle damage. In this study, used the muscle laceration protocol was used, which is associated with extensive muscle fibre damage, intense inflammation and repair through the deposition of fibrous connective tissue. Initially, this process is linked to impaired tissue functionality. The present results demonstrated beneficial effects of treatment as early as the seventh day using the oral formulation of Angiotensin‐(1‐7) included in hydroxypropyl β‐cyclodextrin [HPβ‐CD‐Ang‐(1‐7)] on the skeletal muscles of rats that experienced muscle laceration injury. These effects included the maintenance of physical performance, inflammation control and reduced connective tissue deposition during the muscle repair.

The decrease in body mass observed in animals treated with HPβ‐CD‐Ang‐(1‐7) from the fourth week onwards of treatment, might have been mediated by Ang‐(1‐7)‐induced thermogenesis in brown adipose tissue [30]. In studies with mice was demonstrated that Ang‐(1‐7) can stimulate caloric expenditure by increasing mitochondrial respiration, contributing to decreased body fat mass [30]. Similar findings were also reported in murine models and was indicated that chronic Ang‐(1‐7) infusion in an obesity model was associated with reduced total body weight gain and an increase in lean body mass, indicating the peptide's role in reducing adipose tissue mass [31, 32]. The present results align with previous findings that showed that chronic treatment with HPβ‐CD‐Ang‐(1‐7) led to changes in adipose tissue profile, including reduced white adipose tissue, increased brown adipose tissue, and improved metabolic parameters [33].

Muscle injuries can be defined based on morphological and physiological indices [34]. Such injuries can often be measured by myofibrillar structure rupture or force production reduction. Decreases in functionality based on physiological criteria or structural rupture using morphological criteria are often sufficient to identify the occurrence of injury [34]. In this study, both reduced treadmill running performance and morphological alterations in the muscle tissues of animals subjected to the laceration injury protocol were identified, indicating that the protocol was efficient.

Recovery of functional capacity is a crucial marker of successful tissue repair in an injured tissue, and it is the objective of previous experimental and clinical therapeutic studies. In this study, animals with laceration injuries treated with the oral formulation HPβ‐CD‐Ang‐(1‐7) demonstrated improved performance, including higher speed and fewer failures in the treadmill running test. However, this improved physical performance was not observed in animals treated with placebo. Studies on skeletal muscle suggest evidence of SRA component expression in this tissue, hinting at a potential local action of the Ang‐(1‐7) [9] and it was also demonstrated that Ang‐(1‐7) treatment improved muscle endurance in a treadmill running exercise test in animals with Duchenne muscular dystrophy, also improving tissue functionality [29].

In the context of successful repair, controlling the inflammatory phase is critical for repairing injured tissues; conversely, exacerbation of the inflammatory stage disrupts tissue homeostasis and impairs subsequent stages [34]. Previous studies have shown that the SRA plays a significant role in the pathogenesis of inflammatory diseases. Ang‐(1‐7) acts by blocking certain cellular signalling pathways related to tissue changes triggered by Ang II, such as reduced recruitment of inflammatory cells and cytokine release [35, 36]. In the present study, was demonstrated an increased inflammatory infiltrate in the gastrocnemius muscle in response to the damage caused by muscle laceration. However, animals treated with Ang‐(1‐7) had fewer increases in inflammatory cell numbers compared to animals treated with placebo. These findings corroborate previous studies that showed Ang‐(1‐7) infusion and oral treatment normalising skeletal muscle architecture and controlling the inflammatory process in animals with Duchenne muscular dystrophy [29].

Following or concurrently with the end of the inflammatory stages in tissue repair, reparative events occur. These events aim to establish improved structural and functional outcomes in the injured tissue. The tissue's ability to repair is intricately linked to the type of cells involved, initial lesion size, presence of infectious processes and the intensity and duration of the inflammatory phase [37]. In severe injuries such as lacerations, the most common repair involves the deposition of fibrous connective tissue. Although fibrous tissue deposition ends the lesion process, it does not restore previous tissue normality and is associated with decreased local functionality [23].

The release of growth factors (TGF‐β and CTGF) by cells participating in the inflammatory process induces the proliferation of reparative tissue primarily composed of collagen types I and III. In experimental models of muscle injury, initial fibrillar collagen deposition, types III and IV, is followed by remodelling, with replacement by structural collagen, predominantly type I [38]. As the remodelling process advances, the initial collagen (type III) is reabsorbed, and thicker collagen (type I) is produced and organised [39]. Evaluation of type III collagen has been widely used in the literature as a marker for incomplete healing processes [40]. Ultimately, the retraction and increased resistance of fibrotic areas are observed at the end of this process [39].

CTGF is expressed at low levels in normal tissue, but its overexpression leads to deleterious effects, such as tissue damage and fibrosis [41, 42]. Recent observations show that rat myoblasts and myotubes express CTGF in vitro, and its expression is increased by TGF‐β. Moreover, distinct types of fibroblasts increase CTGF expression in response to both mechanical load and exposure to exogenous TGF‐β. In the present study, there was found a significant increase in both type I and III collagen deposition, as well as gene expression of CTGF mRNA in skeletal muscle in response to damage caused by muscle laceration. Notably, the most significant findings were the reduced collagen deposition and CTGF mRNA gene expression in animals with lacerations treated for 7 and 21 days with Ang‐(1‐7) compared to the placebo. These results align with studies demonstrating the effects of both Ang‐(1‐7) treatment and AT1 receptor blockers (losartan) in reducing muscle tissue fibrosis [14]. Losartan treatment in C57BL/10 mice demonstrated reduced type III and I collagen, as well as other extracellular matrix proteins [14]. Furthermore, that study emphasised the significance of the AT1 receptor blocker (losartan) as a potential anti‐fibrotic agent, especially due to its inhibition of CTGF. It was also demonstrated that systemic infusion and oral treatment with Ang‐(1‐7) normalised muscle tissue architecture, decreased local fibrosis, and improved function in animals with Duchenne muscular dystrophy [18].

Previous studies have demonstrated the involvement of the SRA in the pathogenesis of fibrotic diseases. Most pro‐inflammatory and fibrogenic actions of the SRA appear to be because of the ACE‐AngII‐AT1 axis [43, 44, 45]. On the other hand, the ACE2‐Ang‐(1‐7)‐Mas axis has been repeatedly mentioned in recent decades, and the RAS activity and actions depend on the balance between the ACE‐AngII‐AT1 and ACE2‐Ang‐(1‐7)‐Mas axes [46, 47, 48]. Moreover, a series of landmark studies were crucial in supporting the new concept that the ACE2‐Ang‐(1‐7)‐Mas axis could counter‐regulate the role and functions of AngII, exerting antifibrogenic and antiproliferative actions [17, 18, 29]. More recently, it was demonstrated that Ang‐(1‐7) treatment not only attenuated inflammation but also reduced fibrous connective tissue deposition in skeletal muscle induced by eccentric contraction [12].

To evaluate the role of Ang‐(1‐7) in blocking fibrogenic pathways, the effect of oral Ang‐(1‐7) treatment on Smad7 gene expression was assessed. An inhibitory protein that prevents Smad2/3 and Smad4 complex phosphorylation and formation, proteins from the Smad family are notably regulated by high CTGF expression. The results demonstrated higher Smad7 gene expression in the laceration injury group treated with Ang‐(1‐7) through PCR analysis. Taken together, these results suggest that one of the mechanisms by which Ang‐(1‐7) reduces muscle tissue fibrosis is through blocking the CTGF/Smad pathway. These findings align with a previous study that reported that Ang‐(1‐7) treatment reduced the expression of collagen types I and III, as well as the inhibition of signalling pathways controlled by TGF‐β/Smad in animals with Duchenne muscular dystrophy [18]. The same was observed by another study, demonstrating that Ang‐(1‐7) included in hydroxypropyl β‐cyclodextrin reduced the expression of fibrosis markers (TGF‐β and type I collagen) in an animal model of myocarditis [27]. Additionally, some studies have shown that elevated TGF‐β levels and reduced Smad7 levels are often present in tissues with uncontrolled fibrotic responses. Thus, Smad3 inhibition through Smad7 overexpression drastically reduced fibrotic responses in kidney, lung and liver animal models, indicating an important antifibrotic effect of Smad7 by antagonising the TGF‐β/Smad3 signalling pathway [49, 50, 51, 52].

**FIGURE 5:**
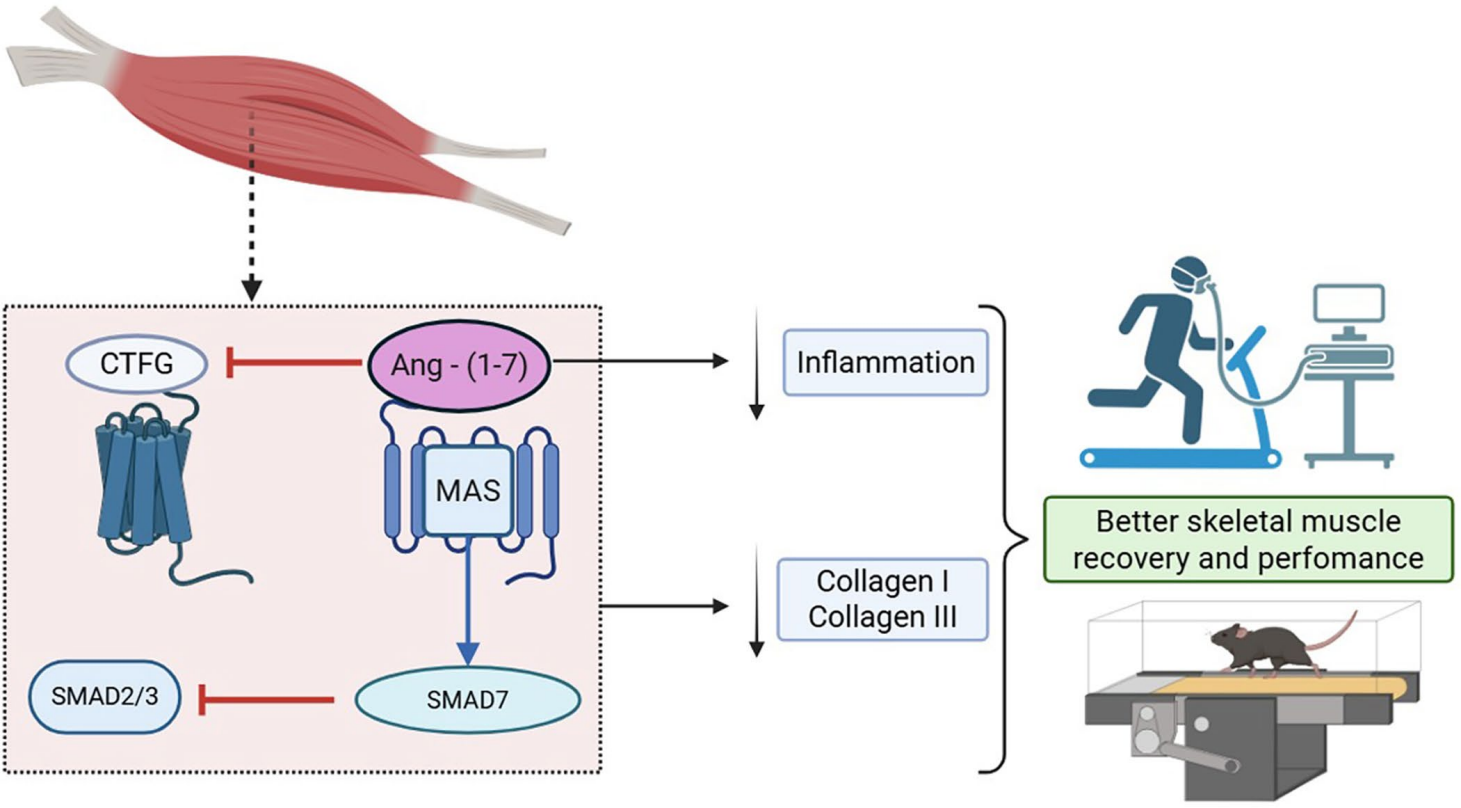
Proposed anti‐fibrotic mechanism of oral HPβ‐CD Angiotensin‐(1‐7) treatment. Ang‐(1‐7) downregulates pro‐fibrotic CTGF expression while upregulating anti‐fibrotic SMAD7, ultimately reducing collagen I and III deposition and promoting better skeletal muscle recovery after injury. The figure nicely illustrates the molecular pathway from the muscle injury (top) through the cellular signalling cascade (dotted box) to the therapeutic outcomes (right side with the exercise figure and research bench).

Taken together, our findings support a mechanistic model in which oral administration of the HPβ‐CD‐Angiotensin‐(1‐7) formulation exerts anti‐fibrotic effects in skeletal muscle following laceration injury. Specifically, Ang‐(1‐7) downregulates pro‐fibrotic markers such as CTGF, while upregulating anti‐fibrotic mediators including SMAD7. These molecular actions contribute to reduced deposition of extracellular matrix components, notably collagen I and III, thereby facilitating structural remodelling and functional recovery of the injured muscle. This proposed mechanism, illustrated in Figure 5, highlights the therapeutic potential of Ang‐(1‐7) in promoting skeletal muscle repair and improving performance, consistent with evidence from both preclinical and clinical studies.

### 4.1 Future Perspectives

Future studies are warranted to comprehensively evaluate the effects of Ang‐(1‐7) on skeletal muscle. It will be important to measure muscle tissue weight from several anatomical sites to assess potential regional differences. Additionally, dual‐energy X‐ray absorptiometry (DXA) should be employed to determine the percentage of fat‐free mass. The quantification of both pro‐ and anti‐inflammatory interleukins, including IL‐8, IL‐10 and CCL2, is also recommended. Finally, functional evaluations, such as isolated muscle preparations for contractile performance testing, should be incorporated to provide deeper insight into the therapeutic potential of Ang‐(1‐7).

## 5 Conclusion

The oral treatment with the HPβ‐CD Angiotensin‐(1‐7) formulation holds significant importance in managing muscle injuries, particularly those involving fibrosis. Angiotensin‐(1‐7) plays a pivotal role in controlling the inflammatory phase, exerting influence over fibrosis production. This influence is manifested through the reduction of pro‐fibrotic factors such as CTGF and the increase in Smad7 proteins, which counter‐regulate fibrogenic pathways. Consequently, the observed outcome is a diminished deposition of extracellular matrix components (collagen types I and III), leading to the preservation of tissue functionality.

## Author Contributions

**Nádia Lúcia Totou:** data curation (equal), formal analysis (equal), investigation (equal), methodology (equal), validation (equal), visualization (equal), writing – original draft (equal). **Ana Maria Sampaio Rocha:** data curation (equal), formal analysis (equal), investigation (equal), validation (equal), visualization (equal). **Samara Silva de Moura:** data curation (equal), formal analysis (equal), investigation (equal), methodology (equal), validation (equal), visualization (equal). **César Henrique Pereira:** data curation (equal), methodology (equal), validation (equal), visualization (equal). **Fabricio Sampaio Coelho:** methodology (equal), validation (equal), visualization (equal). **Douglas Daniel Dophine:** methodology (equal), validation (equal), visualization (equal). **Daniel Barbosa Coelho:** funding acquisition (equal), resources (equal). **Emerson Cruz de Oliveira:** software (equal), supervision (equal), writing – original draft (equal), writing – review and editing (equal). **Robson Augusto Souza dos Santos:** conceptualization (equal), funding acquisition (equal), project administration (equal), resources (equal), supervision (equal), writing – review and editing (equal). **Lenice Kappes Becker:** conceptualization (equal), funding acquisition (equal), project administration (equal), resources (equal), supervision (equal), writing – original draft (equal), writing – review and editing (equal). **Wanderson Geraldo de Lima:** conceptualization (equal), funding acquisition (equal), project administration (equal), resources (equal), supervision (equal), writing – original draft (equal), writing – review and editing (equal).

## Conflicts of Interest

The authors declare no conflicts of interest.

## Data Availability

The data supporting the findings reported here are available from the corresponding author, upon reasonable request.
